# Microbial Consortium with High Cellulolytic Activity (MCHCA) for Enhanced Biogas Production

**DOI:** 10.3389/fmicb.2016.00324

**Published:** 2016-03-15

**Authors:** Krzysztof Poszytek, Martyna Ciezkowska, Aleksandra Sklodowska, Lukasz Drewniak

**Affiliations:** Laboratory of Environmental Pollution Analysis, Faculty of Biology, University of WarsawWarsaw, Poland

**Keywords:** lignocellulosic biomass, cellulose, hydrolysis, anaerobic digestion, microbial consortium

## Abstract

The use of lignocellulosic biomass as a substrate in agricultural biogas plants is very popular and yields good results. However, the efficiency of anaerobic digestion, and thus biogas production, is not always satisfactory due to the slow or incomplete degradation (hydrolysis) of plant matter. To enhance the solubilization of the lignocellulosic biomass various physical, chemical and biological pretreatment methods are used. The aim of this study was to select and characterize cellulose-degrading bacteria, and to construct a microbial consortium, dedicated for degradation of maize silage and enhancing biogas production from this substrate. Over 100 strains of cellulose-degrading bacteria were isolated from: sewage sludge, hydrolyzer from an agricultural biogas plant, cattle slurry and manure. After physiological characterization of the isolates, 16 strains (representatives of *Bacillus, Providencia*, and *Ochrobactrum* genera) were chosen for the construction of a Microbial Consortium with High Cellulolytic Activity, called MCHCA. The selected strains had a high endoglucanase activity (exceeding 0.21 IU/mL CMCase activity) and a wide range of tolerance to various physical and chemical conditions. Lab-scale simulation of biogas production using the selected strains for degradation of maize silage was carried out in a two-bioreactor system, similar to those used in agricultural biogas plants. The obtained results showed that the constructed MCHCA consortium is capable of efficient hydrolysis of maize silage, and increases biogas production by even 38%, depending on the inoculum used for methane fermentation. The results in this work indicate that the mesophilic MCHCA has a great potential for application on industrial scale in agricultural biogas plants.

## Introduction

Plant biomass containing large amounts of lignocellulose, known as lignocellulosic biomass, is a substrate commonly used in agricultural biogas facilities. Although lignocellulose is a good source of energy and substrates for biofuel production, utilization of this material is limited due to its low degradability. As hydrolysis is the first step of anaerobic digestion, preliminary degradation of lignocellulose is crucial for efficient bioconversion of biomass to biofuels (Sun and Cheng, 2002; O’Sullivan and Burrell, 2007). Low rate of hydrolysis of lignocellulose results in the slowdown of the entire process of plant biomass degradation, thus leading to the reduction of the efficiency of fermentation. On the other hand, excessive hydrolysis of plant substrates can lead to the accumulation of intermediate products, which can contribute to system overload and deceleration (or complete inhibition) of the activity of microorganisms carrying out the final stages of biofuel production. Therefore, lignocellulosic material requires special preliminary treatment before further processing (Taherzadeh and Karimi, 2008), which may be carried out by physical, chemical and biological methods (Sanchez, 2009).

Among the recently developed methods of lignocellulose degradation are biological pre-treatment systems, involving the use of either (i) pure, isolated cellulolytic enzymes or enzyme complexes or (ii) microorganisms, mainly bacteria and fungi, which produce such enzymes *in situ* (Maki et al., 2009). The enzymatic method, however, has several restrictions as (i) it can be affected by many factors including the type of substrate, pre-treatment time, system configuration and environmental conditions (e.g., pH, temperature), which have to be carefully controlled; (ii) the prepared enzymes must be regularly added to the working biogas plant. Thus, the use of free enzymes may be less efficient and effective than cultivation of microbial consortia, which stably and continuously produce compounds responsible for the degradation of lignocellulose (Parawira, 2011).

Microorganisms designated for use in biogas production can be isolated from various ecological niches, including soil, agricultural residues, manure or rumen of animals, where they often form specialized consortia, capable of degrading lignocellulose. Although practical application of individual strains is possible, many studies reported that using a mixture of several isolated microorganisms is more effective (Odom and Wall, 1983; Lewis et al., 1988). As the efficiency of biogas production by specified, naturally occurring communities of lignocellulolytic bacteria may be limited by the changes in physico-chemical conditions during the pre-treatment of substrates (lignocellulose), synthetic consortia, which tend to be more robust to environmental fluctuations are developed (Brenner et al., 2008). Biologically stable and controllable consortia of microorganisms with a high hydrolytic activity seem to be most valuable (Zuroff et al., 2013). Many hydrolytic consortia used in biofuel production consist mainly of species of fungi, especially white rot fungi (e.g., *Coriolus versicolor*, *Fusarium* sp., *Phanerochaete chrysosporium*, *Ceriporiopsis subvermispora*, *Cyathus stercoreus*, and *Pleurotus ostreatus*). They secrete unique ligninolytic enzymes, which, though they are effective only for delignification, increase enzymatic degradability of lignocellulosic biomass (Keller et al., 2003; Wan and Li, 2010; Pinto et al., 2012). Other applicable microbial consortia usually consist of 2-4 strains, among which bacteria of the genus *Clostridium* are most common. Mixed cultures of cellulolytic and non-cellulolytic bacteria are also used. These are very often cultivated under strictly anaerobic conditions, using pre-reduced media. Attempts were made to remove this inconvenience, e.g., by using strains with lower requirements and showing higher tolerance to oxygen.

Nowadays, the research focused on aerotolerant communities has been somewhat limited. There is also a relatively small number of recent studies describing cellulolytic consortia cultivated in natural media. A structurally stable, thermophilic consortium (MC1) was isolated from composting materials (sugarcane dregs, chicken feces, dried straw, pig feces, and cattle feces) and it was found to be capable of degrading various cellulosic materials such as rice straw, corn stalk, paper, cotton, and cassava residues (Haruta et al., 2002; Guo et al., 2011). Wongwilaiwalin et al. (2010) developed another thermophilic, lignocellulose-degrading microbial consortium (MC3F) from sugarcane bagasse compost, which can degrade bagasse, rice straw, corn stover and industrial eucalyptus pulp sludge. Kato et al. (2005) obtained and characterized a functional and structurally stable mixed culture (designated SF356) consisting of five bacterial strains (*Clostridium straminisolvens* CSK1, *Clostridium* sp. FG4, *Pseudoxanthomonas* sp. M1-3, *Brevibacillus* sp. M1-5, and *Bordetella* sp. M1-6). Another microbial community, capable of degrading wheat straw, was successfully isolated from plant litter and soil and named WSD-5 (Wang et al., 2011).

Most of the current studies in the field of biofuel production are focused on the search for hydrolytic consortia, which, by the degradation of lignocellulosic biomass, increase the efficiency of anaerobic digestion, and thus the process of methane fermentation. Moreover, the applicable microorganisms should be: (i) stable, (ii) relatively cheap (iii) universal (with a broad spectrum of activity) and (iv) resistant to the changing environmental conditions. It is, however, difficult to fulfill all the above requirements.

This study is a continuation of research on the use of microbial consortia for degradation and anaerobic digestion of plant matter to obtain biofuel. It is also an attempt to answer and solve particular problems encountered during biogas production, so as to increase the efficiency of the process. The objectives of this investigation are the following: (i) selection and characterization of hydrolytic bacteria from different habitats, adopted to various and changing environmental conditions, (ii) construction of a Microbial Consortium with High Cellulolytic Activity, called MCHCA from the isolated microorganisms, which will increase degradation of maize silage, and (iii) simulation of the biogas production in a system similar to those used in agricultural biogas plants, i.e., with separate and independent hydrolysis and methane fermentation steps, using the developed consortium for the pretreatment of the substrate.

## Materials and Methods

### Media and Growth Conditions

Carboxymethylcellulose (CMC) medium (Bushnell and Haas, 1941) was used for isolation of hydrolytic bacteria, estimation of endoglucanase activity and optimization of culture conditions. CMC-Congo Red agar plates (Hendricks et al., 1995) were used for screening for hydrolytic activity. All the isolated strains and the control *Escherichia coli* strain were routinely grown in Luria-Bertani (LB) medium (Sambrook and Russell, 2001) at 30°C and 37°C, respectively. For the strains carrying pGEM-T Easy vector, LB was supplemented with ampicillin at the concentration of 100 g/mL.

Maize silage obtained from a farm in Trzebieszow Pierwszy (Poland) was mixed with low-mineral water (Eden Springs, Poland) and used as a substrate for biological pretreatment and anaerobic digestion processes (maize silage-water medium, MSW).

### Isolation of Hydrolytic Bacteria

Hydrolytic bacterial strains were isolated from: (i) a hydrolyzer from an agricultural biogas plant located in Miedzyrzec Podlaski (Poland), where maize silage is used as a substrate, (ii) cattle slurry (CS) and (iii) manure from farms in Niemglowy and Trzebieszow Pierwszy (Poland). Sterile CMC medium was inoculated with 0.5% of dry weight (DW) of the aforementioned samples. The cultures were incubated for 96 h at 30°C with shaking at 120 rpm. After incubation, diluted cultures were plated on CMC-Congo Red agar plates and incubated for 96 h at 30°C to screen for hydrolytic bacteria. Strains producing a clear zone on CMC-Congo Red agar plates were selected for further assessment of endoglucanase activity.

### Determination of the Optimal Culture Conditions and Screening for Cellulolytic Activity of Bacterial Isolates by Endoglucanase Activity Assay

The isolated pure cultures first were cultivated on modified CMC medium supplemented with different concentrations of CMC: 0.5, 1, 1.5, and 2%. Further optimization included culturing of the strains on 1% CMC medium under different growth conditions: (i) pH 4, 7, 10 and (ii) the temperature of 23°C, 30°C, 37°C, and 45°C. The optical density and endoglucanase (carboxymethylcellulose, CMCase) activity of the cultures in each variant was measured every 24 h. Determination of the optimal culture conditions was performed in three independent replicates.

Endoglucanase activity was determined by a method, recommended by IUPAC Commission of Biotechnology (Ghose, 1987) based on the assessment of the amount of reducing sugars (equivalent of glucose) generated during enzymatic hydrolysis of CMC, carried out by the tested strains grown on minimal medium with 1% CMC. After cultivation on CMC medium, the cultures were centrifuged at 7000 rpm for 15 min at 4°C. The obtained supernatant was used as a crude enzyme solution for the estimation of CMCase activity. The enzyme assay was carried out by incubating the 0.5 ml enzyme solution with 0.5 ml 2% CMC in 0.05 mM sodium citrate buffer (pH 4.8) at 50°C for 30 min. After incubation, the reaction was terminated by the addition of 3 ml of 3,5-dinitrosalicylic acid (DNS) reagent to 1 ml of the reaction mixture. Reducing sugars concentration was estimated spectrophotometrically, by measuring the absorbance at 540 nm. The enzymatic activity of endoglucanase was expressed in international units (IU), where one unit of enzymatic activity is defined as the amount of enzyme that releases 1 μmol of reducing sugars (measured as glucose equivalent) per ml per minute.

### DNA Manipulations and PCR Conditions

Total DNA from pure cultures of strains was isolated using rapid alkaline extraction procedure (Gathogo et al., 2003). Amplification by PCR was performed with a thermocycler (Biorad) using synthetic oligonucleotides and *Taq* polymerase with the supplied buffer (Qiagen), and an appropriate template DNA. For the amplification of bacterial 16S rRNA gene fragments the primer pair 27f and 1492r (Lane, 1991) was used. The PCR products were analyzed by electrophoresis on 0.8% agarose gels, purified with a PCR Purification Kit (Qiagen) and cloned into the pGEM-T Easy vector (Promega). DNA sequencing was performed at the DNA Sequencing and Oligonucleotide Synthesis Laboratory at the Institute of Biochemistry and Biophysics, Polish Academy of Sciences (IBB PAN) using an ABI3730DNA analyzer (Applied Biosystems).

### Nucleotide Sequence Accession Numbers and Strain Deposits in Culture Collection

The 16S rDNA gene sequences were compared with other bacterial sequences deposited in the GenBank database using the BLAST program provided by the NCBI. The sequence data determined in this study were submitted to GenBank and published with the following accession numbers: KJ777134 (KP1), KJ777137–KJ777143 (KP4–KP10), KJ777145–KJ777147 (KP12–KP14), KJ777149–KJ777152 (KP16–KP20), KJ777154 (KP22).

All the strains have been deposited as five mixtures of bacterial strains in the Polish Collection of Microorganisms (PCM) of the Institute of Immunology and Experimental Therapy, Polish Academy of Sciences, Wroclaw, Poland: *Bacillus* sp. KP7, KP20 and *Ochrobactrum* sp. KP8 (a mixture deposited under the no. B/00064); *Providencia* sp. KP14, *Bacillus* sp. KP6 and KP16 (a mixture deposited under the no. B/00065); *Bacillus* sp. KP4, KP5, KP17 and KP22 (a mixture deposited under the no. B/00066); *Providencia* sp. KP10, *Bacillus* sp. KP1 and KP19 (a mixture deposited under the no. B/00067); *Ochrobactrum* sp. KP13, *Bacillus* sp. KP9 and KP12 (a mixture deposited under the no. B/00068).

### Determination of the Total Cell Count

The number of cells in bacterial cultures was determined by staining diluted samples with DAPI (6-diamidino-2-phenylindole) dye at the concentration of 1 μg/ml. Following 20 min of incubation in the dark, the samples were set onto 0.2 μm black polycarbonate membrane by filtration and dried.

The detection of the stained microbial cells was performed under an Nikon Eclipse 80i fluorescence microscope (Japan) at a magnification of 600×, using the filter set WU for DAPI detection. Images of the samples were taken with a Nikon digital camera.

### Hydrolysis of Maize Silage

The MCHCA consortium used in this study was prepared from overnight pure cultures of the isolated cellulolytic bacteria carried out on CMC medium, mixed in equal proportions. The cell count was determined using fluorescence staining with DAPI dye, and set at 10^8^ cells/mL before application.

The effect of biological pretreatment of maize silage by MCHCA and by the pure cultures of strains showing the highest endoglucanase activity (KP16, KP19, KP22) was investigated. The substrate was homogenized in a blender and mixed with low-mineral water. The resulting pulp, containing 3% DW maize silage, was inoculated with 10% (v/v) of the bacterial cultures, to reach the cell density of approximately 10^10^ cells/mL. The pH was adjusted to 7.2 using sodium bicarbonate.

The cultures were carried out for 72 h at 30°C, and samples were collected at the beginning of the experiment and every 24 h. The following parameters were assessed for each sample: pH, soluble chemical oxygen demand (sCOD), volatile fatty acids (VFAs) concentration and the total reducing sugar (glucose) released due to cellulolytic activity of bacteria. The VFAs concentration and sCOD were determined using Nanocolor^®^ kits (Machery-Nagel GmbH, Germany). The total content of reducing sugar was determined by the method developed by Miller (1959). The experiment was performed in three independent replicates.

### Simulation of the Anaerobic Digestion Process

The anaerobic digestion was carried out in a 1 L glass lab-scale bioreactor connected to a Tedlar gas sampling bag (Sigma, Germany) The reactor was supplied with CS as methanogenic inoculum (75% v/v; culture density of approximately 10^10^ cells/mL) and maize silage (1% DW) as a substrate. Low-mineral water was then added to the total volume of 900 mL and the pH of the resulting suspension was adjusted to 7.2 using sodium bicarbonate. The cultures were purged with N_2_ for 5 min to remove the oxygen, and they were incubated at 37°C, for 21 days.

**FIGURE 1:**
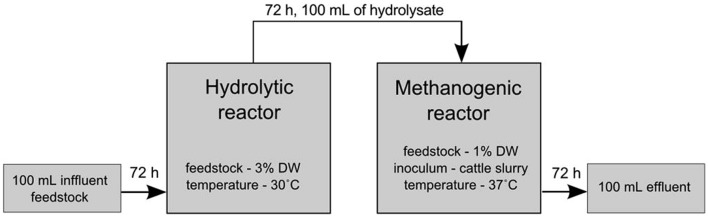
**Schematic presentation of the experimental set-up of the two-phase sequencing reactor of anaerobic digestion.** Hydrolysis was carried out at 30°C for 72 h and anaerobic digestion was performed in a separate reactor at 37°C for 21 days.

After 72 h, 100 mL of the culture were collected into bottles and supplemented with a fresh portion of either untreated (control) or pretreated maize silage. The pretreatment of maize silage was carried out in a continuous manner with the use of the MCHCA consortium or single strains described above. After 72 h of pretreatment, 25% of the volume of the culture was transferred via syringe from the hydrolysis phase to methanogenesis phase. The cultures carrying out hydrolysis were complemented with a fresh portion of 3% DW maize silage and mineral water. The scheme of the experiment is shown in **Figure 1**. All simulations of the anaerobic digestion process were performed in three independent replicates.

To monitor the anaerobic digestion process, the following parameters were determined: the volume and composition of the biogas, VFAs concentration, sCOD and the pH. The DW samples were dried in an oven at 105–110°C for 24 h and analyzed as described in American Public Health Association [APHA] (1998) Standard Methods. Biogas production was monitored by measuring the volume of water displaced from a sealed bottle and collected in a graduated cylinder (Esposito et al., 2012). Methane content was analyzed by gas chromatography with mass spectrometry (GC-MS) using an Agilent 7890A gas chromatograph connected to an electron impact ionization source 5975 series MSD (Agilent Technologies, USA).

## Results and Discussion

### Isolation and Identification of Hydrolytic Bacteria

Hydrolytic bacteria were isolated from a hydrolyzer tank from an agricultural biogas plant in Miedzyrzec Podlaski (Poland), CS and manure collected from farms in Niemglowy and Trzebieszow (Poland). Among the 100s of isolates producing hydrolytic zones on 1% CMC-Congo Red agar plates, sixteen strains (**Table 1**) showed elevated endoglucanase activity. After 72 h of incubation, these strains produced clear zones reaching from 15 to 44 mm in diameter, with the highest result for the strain KP1. The diameters of the zones are similar to those reported by Gupta et al. (2012), and Liang et al. (2014), 28–50 mm and 20–30 mm respectively, obtained for cellulolytic bacteria isolated from guts of termite, caterpillar, bookworm and snail, as well as organic-rich soil. The taxonomic analysis based on the partial 16S rDNA sequences (∼1.4 kbp) showed that most of the isolates described in this study belong to the *Bacillus* genus (12 strains) (**Table 1**). The other strains were classified to the *Ochrobactrum* (2 isolates) and *Providencia* (2 isolates) genera (**Table 1**). This result is consistent with the literature, where cellulolytic bacteria, mainly belonging to the following genera: *Cellulomonas, Clostridium, Bacillus, Paenibacillus, Thermomonospora, Ruminococcus, Bacteriodes, Erwinia, Acetovibrio, Microbispora, Pseudomonas* and *Streptomyces* are described. These cellulolytic bacteria were isolated from different environmental niches, e.g.: sewage sludge, CS and manure, various types of soil and compost samples (Saratale et al., 2010; Okeke and Lu, 2011).

**Table 1:** Identification and characterization of the isolated bacteria.

Isolate number (accession number)	CMCase activity [IU/ml]	Maximum clearing zone on CMC- Congo Red agar plates [mm]	Phylogenetic position (based on the analysis of the 16S rDNA sequence)	
			
			Best match in GenBank database (accession number)	Identity (%)
KP1 (KJ777134)	0.354	44	*Bacillus licheniformis* strain Pb-HK09002 (HM006898.1)	99
KP4 (KJ777137)	0.298	27	*Bacillus pumilus* strain SAFR-032 (NR_074977.1)	98
KP5 (KJ777138)	0.210	15	*Bacillus pumilus* strain Jo2 (KF734912.1)	99
KP6 (KJ777139)	0.330	26	*Bacillus pumilus* strain 43 (KF923453.1)	99
KP7 (KJ777140)	0.435	22	*Bacillus altitudinis* strain EH36 (GU339265.1)	96
KP8 (KJ777141)	0.218	17	*Ochrobactrum* sp. c261 (FJ950617.1)	98
KP9 (KJ777142)	0.472	15	*Bacillus* sp. B2066 (JX266376.1)	99
KP10 (KJ777143)	0.243	16	*Providencia* sp. strain FFA6 (JN092794.1)	99
KP12 (KJ777145)	0.280	29	*Bacillus pumilus* strain 38 (KF923448.1)	99
KP13 (KJ777146)	0.248	14	*Ochrobactrum sp.* c261 (FJ950617.1)	98
KP14 (KJ777147)	0.384	15	*Providencia* sp. strain FFA6 (JN092794.1)	95
KP16 (KJ777149)	0.591	33	*Bacillus aerius* strain RGS230 (KC469617.1)	99
KP17 (KJ777150)	0.255	21	*Bacillus subtilis* isolate SCS-3 (EU257431.1)	99
KP19 (KJ777151)	0.392	25	*Bacillus pumilus* strain 38 (KF923448.1)	99
KP20 (KJ777152)	0.498	38	*Bacillus pumilus* strain 38 (KF923448.1)	99
KP22 (KJ777154)	0.434	35	*Bacillus licheniformis* strain AnBa7 (AY887129.1)	99


### Endoglucanase Activity

**FIGURE 2:**
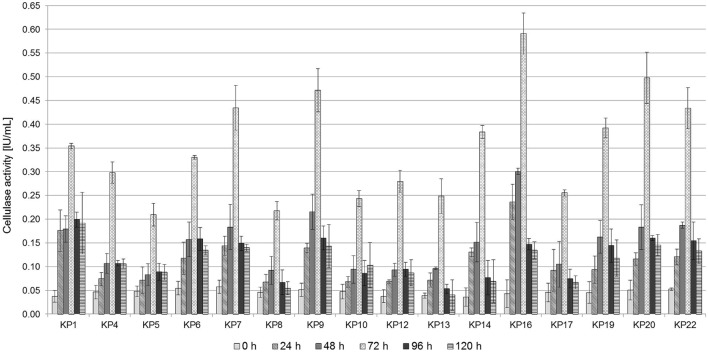
**Carboxymethylcellulose (CMC) activity in the cell-free culture supernatants of the isolated strains.** Bacterial cells were grown in 1% CMC liquid medium (pH 7.0) for 5 days at 30°C. Culture supernatants were assayed for CMCase activity every day. Mean values from triplicate experiments are presented.

The diameter of the hydrolyzing zone produced by bacteria on CMC-Congo Red medium may not accurately reflect their true cellulase activity (it is a semi-quantitative method). For this reason endoglucanase activity of the selected strains was quantitatively assessed using the dinitrosalicyclic acid method. Bacterial cultures were carried out for 120 h on 1% CMC medium, and the endoglucanase activity was measured every 24 h. It was revealed that the highest endoglucanase activity was observed after 72 h of culturing for all strains and it was in the range between 0.21 IU/mL, for the strain KP5, and 0.59 IU/mL, for the strain KP16 (**Figure 2**). After that time, a decrease in the hydrolytic activity and the concentration of glucose was observed, which may be associated with the effective hydrolysis of CMC. The glucose formed in the reaction culture is rapidly used as a metabolic substrate. A similar trend and the value of CMCase activity was observed for all the tested strains and for the tested microbial consortium.

Several studies have been carried out to investigate the endoglucanase activity of similar aerobic bacteria. For instance, the maximum CMCase activity of *Bacillus subtilis* AS3 was found to be 0.43 IU/mL (Deka et al., 2011). Gupta et al. (2012) isolated several cellulose-degrading bacteria exhibiting CMCase activities in the range of 0.162–0.400 IU/mL. Similar results were also reported for *Acinetobacter anitratus* (0.48 IU/mL) (Ekperigin, 2007). Irfan et al. (2012) demonstrated that the isolated *Cellulomonas* sp. ASN2 strain showed CMCase activity at a level of 0.451 IU/ml. The results obtained in this study show that the most active isolates are characterized by a higher hydrolytic activity (**Figure 2**).

However, it must be emphasized that it is very difficult to compare endoglucanase activity of various bacteria and determine whether its value is high or low, as the individual strains described in the literature were cultivated under different conditions.

### Growth Conditions

The optimal growth conditions for all the selected strains were determined. The analyses performed on CMC medium showed that all of the tested bacteria were capable of growth at a broad range of temperatures (22–45°C) and pH (4–10), and regardless of the conditions, effective cellulose decomposition was observed (**Table 2**).

**Table 2:** Growth characteristic of the isolates.

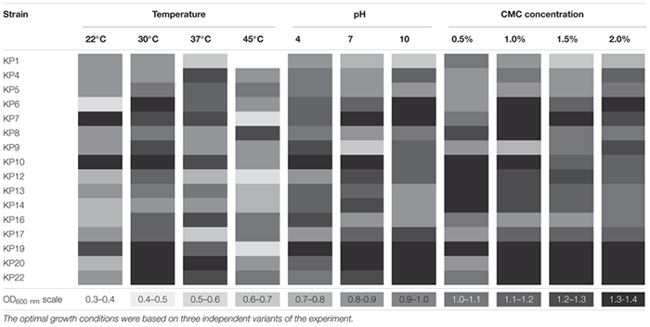

*The optimal growth conditions were based on three independent variants of the experiment.*

The optimum temperature for most of the isolates (nine strains) was 30°C, (**Table 2**), and KP8 was the only strain that could be classified as moderately thermophilic (showing optimum growth at 45°C). The highest growth for most of the strains (8) was observed at pH 7. Only a few strains preferred slightly acidic (KP1, KP5, KP9, KP16) or alkaline pH (KP4, KP6, KP17, KP22) (**Table 2**). The changes in the concentration of CMC in the range of 0.5–2% did not significantly affect the growth of the tested strains, and the highest growth rate was observed in media supplemented with 1 and 2% of CMC (**Table 2**).

Based on the above findings, it can be concluded that the optimum growth conditions for most of the isolated bacteria were: the temperature of 30°C and pH 7, what is very important in the context of hydrolysis of cellulose. Although there have been diverse reports on the optimal initial pH and temperature for cellulolytic enzymes production, it is assumed that most efficient enzymatic hydrolysis occurs at 30°C and pH 7 (Pradip Saha et al., 2012; Salam et al., 2013).

In contrast to pure, isolated enzymes, which may be deactivated by an abrupt change of the pH from neutral to acidic, acid-tolerant bacteria, such as those described in this work, may quite rapidly adapt to the new conditions and continue to secrete (functional) enzymes. Thus, a wide range of growth conditions of the isolated hydrolytic bacteria may allow for an effective degradation of lignocellulosic material also in case of other, uncontrolled changes in the conditions, which may occur during the biofuel production process.

### Hydrolysis of Maize Silage

**FIGURE 3:**
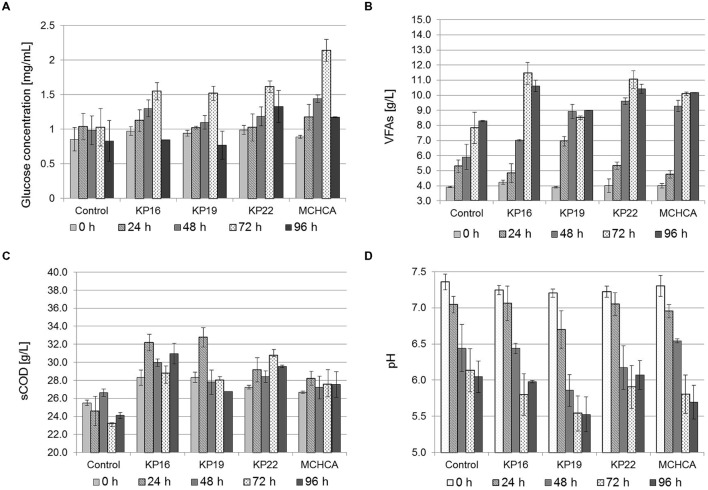
**Solubilization of maize silage by cellulolytic bacteria.** Analysis of the changes in the concentration of: glucose **(A)**, VFAs **(B)**, as well as changes in sCOD **(C)** and the pH **(D)** during anaerobic treatment of maize silage (3% DW) by single strains KP16, KP19 KP22 and MCHCA in MSW medium.

Previous experiments showed that the selected strains have a high endoglucanase activity on synthetic soluble substrates (CMC). Literature data report that endoglucanases are the type of cellulases and some of them also show exoglucanase activity (Sakon et al., 1997; Bayer et al., 1998). In fact, to have true cellulolytic activity, the isolated microorganisms must break down insoluble cellulose (not only CMC). For this reason, a series of experiments on maize silage, a natural substrate utilized in many agricultural biogas plants were carried out using each of the most active strains (KP16, KP19, KP22), and a mixture of all the isolates. The cultures of all the 16 isolates, mixed in equal proportions, constituted a novel consortium called the MCHCA. Hydrolysis of 3% DW maize silage was monitored during 72 h of incubation at 30°C and the effectiveness of the process was estimated based on the level of glucose and VFAs production and changes in sCODs (**Figure 3**).

In all sets of experiments, the hydrolysis of maize silage, expressed as a function of glucose and VFAs production, was observed, and had a similar course in pure cultures and the MCHCA culture (**Figure 3**). After 72 h of incubation, the concentration of VFAs and glucose reached the highest value in single strain cultures (8.52–11.46 g/L of VFAs and 1.520–1.613 mg/L of glucose) and the MCHCA culture (10.10 g/L of VFAs and 2.138 mg/L of glucose). Longer pretreatment resulted in a decrease of VFAs (KP16 and KP22) and glucose concentration in all the cultures (**Figure 3**). Probably, this is a result of the metabolic activity of the indigenous microflora of maize silage, what was confirmed by the results noted in the control, where the highest concentration of glucose was only 1.026 mg/L. In turn, the highest VFAs concentration in the control reached 8.30 g/L after 96 h of incubation. The level of sCOD in the hydrolysate during the pretreatment using MCHCA increased significantly from 26.67 to 27.57 g/L and in single strain cultures (from 27.23–28.33 to 28.03–30.80 g/L) after 24 h of pretreatment. In the control variant, sCOD changed from 25.50 to 26.63 g/L during the same time. The increase of VFAs concentration and sCOD during hydrolysis was mainly due to the degradation of insoluble macro-molecular organic compounds of maize silage. The subsequent decrease of concentration of VFAs and glucose, as well as sCOD, was probably, by the consumption of the soluble metabolites by other groups of microorganisms.

The pH of the hydrolysates decreased during the pretreatment from the initial value of 7.2 to 5.69 and 6.05 for MCHCA and control variants, respectively. The use of strains constituting the MCHCA consortium, which are characterized by a wide range of pH tolerance, potentially allows for the maintenance of hydrolytic activity at a high level during the hydrolysis process even under acidic conditions.

Preparation of mixtures of different microorganisms that increase degradation of natural lignocellulosic material has been previously reported by several researchers. Guo et al. (2010) developed an efficient lignocellulose-degrading composite microbial system (XDC-2) from soil amended with composted agricultural and animal waste. The consortium was composed of mesophilic bacteria belonging to the genera *Clostridium, Bacteroides, Alcaligenes, Pseudomonas*, etc. The content of reducing sugars obtained after the degradation of rice straw and corn stalk was 1.3 g/L and 2.4 g/L, respectively (Guo et al., 2010). Additionally, the pH during the pretreatment rapidly decreased from 7.2 to 5.9, what is consistent with our results. Wang et al. (2013) continued the studies on the XDC-2 consortium and suggested that the decrease in the pH value was due to the production of organic acids (acetic, propanoic and butanoic acids). Yuan et al. (2014) showed that the concentration of VFAs and sCOD increased rapidly during 4 days of pretreatment of corn stalk using the MC1 consortium and it depended on different initial concentration of the substrate. Zhang et al. (2011) constructed a thermophilic microbial consortium capable of increasing the VFAs concentration to up to 10.6 g/L after 72 h of pretreatment of cassava residues and the sCOD to a maximum value of 26.6 g/L after 12 h of pretreatment. In our study, we have observed similar trends with VFAs, as their concentration was high during the first 3 days of the culture, and then it decreased.

The results indicate that the conversion of lignocellulose to soluble products can be regarded as the rate-limiting step during the anaerobic digestion, and the optimum pretreatment time should be equal to that, during which sCOD and the concentration of VFAs reaches the maximum (i.e., 72 h). The obtained results (increased level of glucose, VFAs concentration and sCOD) are a proof of the high hydrolytic activity of MCHCA including cellulolytic activity.

Some of the differences concerning pretreatment efficiency observed between literature data and our findings are the result of using diverse plant matter, e.g., cassava residues, cotton, rice straw, wheat straw or corn stalk as the substrate in various studies. In our research, we have investigated the influence of microorganisms on the degradation of maize silage, one of the most popular substrates used in agricultural biogas plants in Europe. It differs from corn stalk used in other studies, as it is specially preserved by ensiling (Neureiter et al., 2005).

As most of the reports concern thermophilic strains, our results are difficult to compare with literature data. However, the mesophilic strains described in this study show at least the same efficiency of plant matter hydrolysis as the strains that require higher temperatures for optimum growth. Understandably, the degree of degradation of lignocellulosic substrates carried out at 50°C is higher than at lower temperatures, but the use of MCHCA allows for maintaining high levels of hydrolysis even at 37°C, which is a great advantage in terms of energy consumption.

The cooperation and synergism of the isolated microorganisms in the MCHCA consortium enhance their degradation abilities, and the use of a combination of microorganisms is more efficient than using monocultures (pure cultures), especially when the conditions change during the hydrolysis process. Microbial consortia are usually better adapted to pH and temperature changes and tend to show higher resistance to the presence of heavy metals, toxic organic compounds or contamination by other strains. Furthermore, pure cultures were rarely applied to the pretreatment in large-scale biogas production.

### Simulation of the Anaerobic Digestion Process

Pretreatment of maize silage using MCHCA as well as the selected single strains was performed with high efficiency, as elevated levels of glucose and VFAs were observed in the cultures. However, high concentration of VFAs does not always imply efficient production of biomethane, as some fatty acids may inhibit the growth of methanogenic archaea. Therefore, it was important to determine if and how the MCHCA consortium affects the activity of microorganisms involved in anaerobic digestion, and how it affects the efficiency of biogas production. In order to determine the effect of using maize silage pretreated by MCHCA and pure cultures of the selected strains (KP16, KP19, KP22) on the efficiency of biogas production, an experiment was carried out in a two-step system, similar to those used in agricultural biogas plants.

Simulation of the anaerobic digestion process was performed in a semi-continuous mode (**Figure 1**) for 21 days. The subsequent transfer of the hydrolyzed material (every 72 h) to the fermenter was continued throughout the culture. It must be emphasized that the maize silage from the hydrolyzer was the only source of organic compounds for anaerobic digestion. A hypothesis may be drawn that separation of the hydrolysis and anaerobic digestion processes, without recirculation of matter, allows for the stabilization of the cultures in each bioreactor, and it proved to be beneficial for biogas production.

**FIGURE 4:**
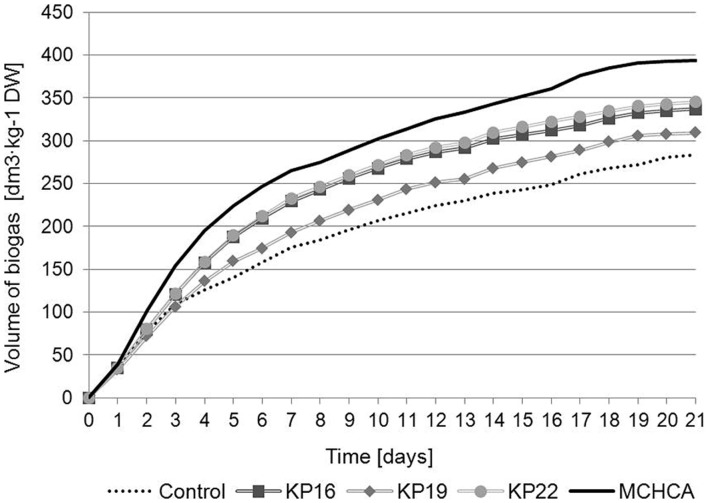
**Cumulative biogas production of untreated and pretreated maize silage during anaerobic digestion**.

**FIGURE 5:**
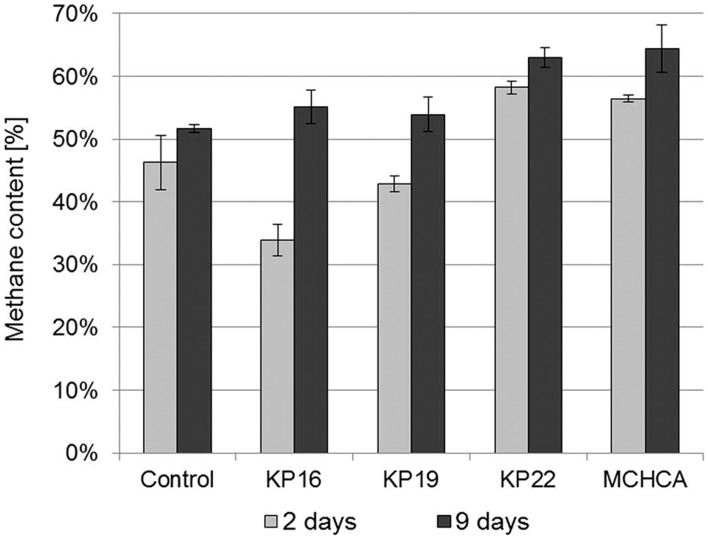
**The methane content in biogas during anaerobic digestion of maize silage – untreated and pretreated by the pure cultures and the MCHCA consortium**.

The cumulative biogas production (CBP) and the content of methane in the produced biogas (CM) revealed that MCHCA significantly increased the efficiency of methanisation of maize silage (**Figures 4** and **5**). The CBP for maize silage treated by MCHCA was 393.365 dm^3^/kg of DW and 336.67 dm^3^/kg of DW, 309.303 dm^3^/kg of DW and 344.925 dm^3^/kg of DW for KP16, KP19, KP22, respectively. The production of biogas in the experiment with untreated maize silage (control variant) was 238.185 dm^3^/kg of DW. The results showed that between days 3 and 21 of the culture the volume of biogas production using maize silage hydrolyzed by MCHCA and single strains, increased by 38 and 16%, respectively (**Figure 4**).

The methane content was the highest after the first 9 days of anaerobic digestion using the maize silage pretreated by MCHCA (increased from 56.46 to 64.41% CH_4_) and by the single strains (increased from 33.93 to 62.95% CH_4_). In contrast, no clearly significant changes were noted for the control culture (change in CH_4_ content from 46.25 to only 51.67%) (**Figure 5**). These results indicate that the accumulation of intermediate products in the maize silage hydrolysate during the pretreatment using MCHCA and single strains is beneficial to the microorganisms carrying out anaerobic digestion.

Yuan et al. (2014) showed that biogas and methane production yields from lignocellulose from municipal solid waste were significantly increased following pretreatment by the MC1 thermophilic microbial consortium. The biogas yields were 404 dm^3^/kg DW after 4 days of treatment of the pretreated waste at the concentration of 2.5%. These values were higher than for the untreated sample (control) and similar to our results (393.365 dm^3^/kg of DW). In addition, the highest percentage of methane content was 50–55% and it was higher than in the control, but lower than that obtained for maize silage samples treated by MCHCA (∼64%). Yan et al. (2012) developed a mesophilic consortium BYND-5, which was used for the pretreatment of rice straw, and which enhanced the total biogas yield by up to 9.3% and the percentage content of methane by up to 10%. Generally, more methane was produced using the treated samples than the untreated samples (Yuan et al., 2014). However, in both cases, methane yield was lower than that obtained from the substrate pretreated by MCHCA.

**FIGURE 6:**
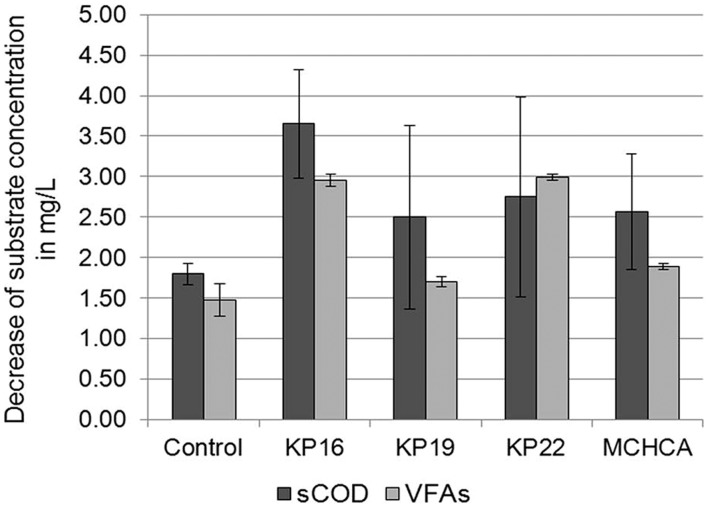
**Decrease of sCOD and VFAs concentration during biogas production (anaerobic digestion) from maize silage**.

The conditions of the anaerobic digestion process are often determined by the physical and chemical parameters. In this study, sCOD and VFAs concentration were determined. The sCOD decreased during the anaerobic digestion after 21 days. A greater decline in sCOD was observed for the KP16, KP22, KP19 and MCHCA variants (from 9.2 to 5.55 g/L, 9.5 to 6.75 g/L, 8.6 to 6.1 g/L, 8.6 to 6.03 g/L, respectively) than in the control (from 7.23 to 5.43 g/L) (**Figure 6**). Furthermore, similar trends were observed for VFAs concentration in both experimental variants. The initial high concentration of VFAs (5.07 g/L for KP16, 5.03 g/L for KP22, 3.975 g/L for MCHCA and 3.805 g/L for KP19) rapidly decreased (to 2.12 g/L for KP16, 2.035 g/L for KP22, 2.08 g/L for MCHCA and 2.1 g/L for KP19) after 21 days. In the control variant, the level of VFAs decreased from 3.93 to 2.45 g/L (**Figure 6**). This is probably caused by the rapid process of glucose conversion to VFAs, and VFAs to other chemical compounds during anaerobic digestion.

High sCOD in the hydrolysates indicated that large amounts of soluble substrates are available for anaerobic digestion. On the other hand, the formed VFAs can play an important role in enhancing the hydrolysis of lignocellulosic material (Yu et al., 2004). Firstly, because the generated compounds are the substrates, which are converted by another group of microorganisms (e.g., syntrophic bacteria) in acetogenesis. Secondly, it was reported that weak acids such as propionic acid, can loosen the structure of lignocellulose, resulting in an improved overall hydrolysis rate, owing to the increased accessibility of the substrate for the enzymes (Cassini et al., 2006). The fact that a correlation was observed between the high hydrolytic activity and high biogas yield during anaerobic digestion suggests that the pretreatment of cellulose/lignocellulose substrates is crucial for promoting biogas productivity and biogas technology development (Haruta et al., 2002; Wongwilaiwalin et al., 2010; Guo et al., 2011).

Our results show that inoculation of maize silage by the new mesophilic microbial consortium MCHCA (constructed from naturally hydrolytic microorganisms isolated from different habitats, adopted to various and changing environmental conditions) can significantly enhance biogas yield (by 38%) and increase the content of methane (up to 64%) during anaerobic digestion.

The strategy of using the two-phase sequencing reactor, described in this study, for anaerobic digestion is typically applied in agricultural biogas plants, where a part of the fermented matter is not recycled for crude nutrient reduction. Recycling of a part of the fermented matter may lead to excessive dilution of the hydrolytic culture in the hydrolyzer and may increase the risk of interspecies competition, leading to a decrease in the efficiency of hydrolysis. This was confirmed in our previous studies (unpublished data), where a system with recirculation of a portion of 1 L fermented material (from a 40-l reactor) was transferred back to the hydrolyser (2-l). In that case an increase of the efficiency of biogas production using pretreated maize silage was observed, but it was almost three-times lower (only 13%) than the result obtained in the system described in this work (38%).

## Conclusion

This study provides an insight on the potential of biological pretreatment of plant matter designated for use in biogas production as an alternative to other, more costly and less environmentally friendly methods. The presented isolates exhibit much higher endonuclease activity than other strains described in the literature, including the reference strain. Moreover, the selected isolates and the MCHCA consortium showed a very high hydrolytic activity (including cellulolytic activity) against maize silage. The use of MCHCA allows for maintaining a high level of hydrolysis even at 37°C, which is a great advantage in terms of energy consumption in industrial processes.

These features make MCHCA suitable for use in agricultural biogas plants to enhance the efficiency of methane production.

## Author Contributions

KP: planned and performed most of experiments, wrote the manuscript; MC: participated in simulation of the anaerobic digestion process; AS: is a head of a group, and was involved in consultation and article correction; LD: is a head of a project; conceived and directed the studies and was involved in consultation and article preparation.

## Conflict of Interest Statement

The authors declare that the research was conducted in the absence of any commercial or financial relationships that could be construed as a potential conflict of interest.
